# Upper Limb Function and Cortical Organization in Youth with Unilateral Cerebral Palsy

**DOI:** 10.3389/fneur.2014.00117

**Published:** 2014-07-04

**Authors:** Anna Mackey, Cathy Stinear, Susan Stott, Winston D. Byblow

**Affiliations:** ^1^Department of Surgery, University of Auckland, Auckland, New Zealand; ^2^Department of Medicine, Centre for Brain Research, University of Auckland, Auckland, New Zealand; ^3^Department of Sport and Exercise Science, Centre for Brain Research, University of Auckland, Auckland, New Zealand

**Keywords:** cerebral palsy, cortical re-organization, upper limb function, hemiplegia

## Abstract

**Aim:** To explore the relationship between motor cortical and descending motor pathway reorganization, lesion type, and upper limb function in youth with unilateral cerebral palsy (CP).

**Methods:** Twenty participants with unilateral CP (mean age 15 ± 3 years; 11 males) completed a range of upper limb functional measures. Structural MRI, diffusion-weighted, and functional MRI were conducted to determine type and extent of brain lesion, descending white matter integrity, and whole-brain activity during affected hand use. Single pulse transcranial magnetic stimulation (TMS) (*n* = 12) was used to examine functional integrity of the corticospinal pathway as well as primary motor cortex intracortical and interhemispheric inhibition from motor-evoked potentials and silent periods.

**Results:** Fractional anisotropy measures within the posterior limb of the internal capsule were a predictor of upper limb function (*R*^2^ = 0.41, *F* = 11.3, *p* = 0.004). Participants with periventricular lesions tended to have better upper limb function [*F*(2, 17) = 42.48, *p* < 0.0001]. Five participants with evidence of cortical reorganization and functional ipsilateral projections to their affected hand had worse upper limb function. Deficits in intracortical and interhemispheric inhibitory mechanisms were found in participants with worse upper limb function (Melbourne Assessment of Unilateral Upper Limb Function: Mann Whitney *p* = 0.02).

**Conclusion:** Neuroimaging and TMS can provide useful information related to hand function of individuals with unilateral CP and may have potential to assist as a predictive tool and/or guide rehabilitation.

## Introduction

Unilateral cerebral palsy (CP) occurs after an insult to the developing brain, resulting in motor and sensory impairments (1). The subsequent upper limb functional deficits can significantly impact on a child’s independence and potentially limit future employment options. A recent systematic review of upper limb therapeutic interventions for children with unilateral CP found a variable effectiveness of current treatments, with a lack of information on how and when to best target interventions (2). This paucity of information is a potential barrier to rehabilitation because it is difficult to appropriately select individuals who are best suited for particular interventions, such as those that are time and labor-intensive, e.g., constraint-induced movement therapy (CIMT).

There is increasing interest in whether neuroimaging can be used to predict response to intervention or therapy in CP (2–4). The timing, size, and location of the lesion influence the topographical presentation of the limbs affected (5), but the determination of the functional outcome for the child is more difficult to predict from structural MRI alone (6). Reorganization of the primary motor cortex (M1) is often evident in unilateral CP. Functional MRI (fMRI) during motor tasks may indicate shifts in activation toward the unaffected hemisphere when using the affected upper limb (7, 8). Motor-evoked potentials (MEPs) from transcranial magnetic stimulation (TMS) of M1 may also reveal reorganization, such as an increased prevalence of MEPs from TMS of the M1 ipsilateral to the affected side (9). The functional consequences of this reorganization are not fully understood.

The aim of this study was to explore the relationship between upper limb function measures in unilateral CP and characteristics of reorganization within and between motor cortices. Anatomical and diffusion-weighted MRI (DW-MRI) was used to assess structural integrity of descending motor pathways, while TMS and fMRI were used to assess the functional integrity and organization of the corticomotor pathways.

## Materials and Methods

The study was approved by the New Zealand Health and Disability Ethics Committee, with written consent obtained from participants and parents, in accordance with the Declaration of Helsinki.

### Participants

We recruited 20 youths from local orthopedic and therapy services. Inclusion criteria were a diagnosis of unilateral CP, age range of 12–25 years, and no history of surgery or botulinum toxin A to the upper limb in the last 12 months. Exclusion criteria were co-morbidities that would limit the participants’ completion of the study and the presence of contraindications to MRI (e.g., metal implants such as teeth braces, which could distort cranial images) or TMS (history of epilepsy).

### Clinical measures

Upper limb function was assessed using the Box and Block Test and the Melbourne Assessment of Unilateral Upper limb Function (MUUL) (10, 11). Note, the MUUL has established psychometric properties for children age 4–15 years; however, it was selected as the most suitable tool to assess unimanual function for the wide age range of participants in this study. The generic functional and upper limb range of movement tasks assessed in this measure (e.g., hand to mouth or forearm supination range of motion) were considered appropriate for older age groups to complete. In addition, five bimanual everyday activities were videoed and scored by an occupational therapist blinded to other measures. Tasks were scored on spontaneous use and amount of assistance of the affected hand during the tasks, using a four-point scale with a maximum score of 30 (12). Self-reported perceptions of difficulty performing daily activities (mainly bimanual) were assessed by using the condition-specific Abilhand-Kids questionnaire (13). The “Kids” version was used for all participants. This version has only been validated in children with CP from 6 to 15 years, but the functional tasks were considered to be appropriate for participants over 15 years and the adult version is not specific to the CP population. The total score was converted to logits for statistical analysis creating an interval score on a continuum (http://www.rehab-scales.org/abilhand-kids.html; last accessed 20/03/2014). Hand function was classified using the Manual Ability Classification System (MACS) (14) and the presence of mirror movements during simple hand tasks was classified on a 0–4 scale, with 0 = no imitative movements through to 4 = movements are equal to that expected of the intended hand, as described by Woods and Teuber (15).

### Magnetic resonance imaging

Magnetic resonance imaging brain scans were acquired with a General Electric, HDx 3 Tesla system. Sagittal and high resolution 3-D T_1_, diffusion-weighted, axial FLAIR, and T_2_ weighted diagnostic imaging was completed to assess the extent of the brain lesion. Structural T_1_-weighted images were acquired with 3D, sagittal sequence of 128 contiguous slices [TR = 11 ms, TE = 4.5 ms, Field of View (FOV) = 240 mm; Inversion time 450 ms and voxel dimensions of 0.47 mm × 0.47 mm × 1.3 mm). Blood oxygenation level-dependent (BOLD) contrast images were obtained using a T_2_ weighted gradient echo EPI sequence of hand motor activity interspersed with rest periods to map hand motor cortical localization (TR = 4 s, TE = 35 ms, FOV = 240 mm, voxel dimensions 3.75 mm × 3.75 mm × 4 mm). Diffusion-weighted imaging was acquired with a spin echo EPI pulse sequence (TR = 8.3 s, FOV = 240 mm, TE = 87.3 ms, and voxel dimensions 0.94 mm × 0.94 mm × 5 mm) with 25 uniformly distributed Stejskal–Tanner motion-probing gradient orientations (*b* = 1000 s/mm^2^) and two *b* = 0 images. All images were reported by a neuroradiologist, blinded to other aspects of the study. Lesions were classified into three categories: cortical–subcortical (C/SC); Periventricular (PV); Malformations (MAL) (16, 17).

During the fMRI experiment, participants completed a self-paced hand grip and release task, using a MR-compatible device. There were three conditions (bimanual, left only, right only) presented in a block design interspersed with rest periods (six cycles per condition; 24 s on and 24 s off). For unimanual trials, the non-active hand was strapped into a resting hand splint. A constant visual cue remained on screen to indicate the rest period. An additional visual cue flashed on every second during the hand motor activity period. Functional images were slice time corrected, realigned, smoothed, and co-registered using FSL/FEAT (http://www.fmrib.ox.ac.uk/fsl/) (18). The difference in BOLD signal between rest and movement condition (bimanual, left, right) was quantified by voxel cluster analysis, using an activation threshold of (*z* = 2.3, *p* < 0.05) bilaterally in the relevant regions of interest (ROI) of primary and premotor cortex and supplementary motor area, derived from the Juelich histological brain atlas (19). A laterality index (LI) was calculated with the equation below to quantify BOLD activation between hemispheres for each ROI during the affected hand movement as LI = [PAV_(CL)_ − PAV_(IL)_]/[PAV_(CL)_ + PAV_(IL)_], where PAV = percentage of active voxels, and IL and CL are ipsilesional (side of the lesion) and contralesional (CL) hemispheres. LI ranges between −1.0 and +1.0, where −1.0 indicates only ipsilesional activity and +1.0 indicates only CL activity. All MR measurements were completed by one experimenter (AM) using a semi-automated pipeline in FSL/FEAT program and manual calculation of LI.

Diffusion-weighted MRI analysis was completed using the FSL/FDT processing pipeline to characterize the white matter tracts within the brain (20). This included distortion correction (eddy current correction), brain extraction, tensor fitting, and establishing scalar files, i.e., Fractional Anisotropy (FA) RGB file. DW–MR imaging data from participants no. 13 and 16 were excluded as excessive head motion during collection resulted in an inability to complete distortion correction or brain masking.

Fractional anisotropy values were determined bilaterally for a region outlining the posterior limb of the internal capsule (PLIC) (21). The PLIC was defined in MRIcro (www.mricro.com) from the level of the anterior commissure to the base of the corona radiata (22) and using the FA RGB files (Note, intra or inter-rater reliability was not completed for determination of the PLIC). An FA asymmetry index (FAAI) was calculated as FAAI = (FA_CL_ − FA_IL_)/(FA_CL_ + FA_IL_). FAAI ranges from −1.0 to +1.0, with positive values indicating reduced FA in the ipsilesional PLIC and 0 indicating symmetrical FA between PLICs (21).

### Transcranial magnetic stimulation

Single pulse TMS of M1 was delivered with a MagStim 200 stimulator (MagStim Company, Dyfed, UK) and a figure-of-8 coil (wing diameter 9 cm) orientated to induce posterior–anterior current flow in M1. MEPs were recorded from surface EMG in bilateral first dorsal interosseus (FDI) and extensor carpi radialis (ECR). The optimal site of stimulation was determined for each target muscle, facilitated by the use of a stereotaxic camera system and Brainsight™ (Rogue Research, Montreal). Rest motor threshold (RMT) was determined in FDI using conventional procedures (23). MEPs were deemed absent if MEP amplitude was less than 50 μV with 100% muscle stimulator output (MSO). Average MEP amplitude and latency were determined from 8 responses to TMS at 120% RMT. The CL hemisphere was stimulated first prior to assessment of responses to stimulation of the ipsilesional hemisphere.

Intracortical and interhemispheric inhibition were examined from measures of cortical silent period (cSP) and ipsilateral silent period (iSP), respectively. The cSP was determined for each hand separately, during wrist extension and a pincer grip contraction at 50% maximal voluntary contraction (24) with TMS at 60% MSO applied to the contralateral “hotspot” for the contracting target muscle. The cSP duration was estimated from the stimulus onset to the return of EMG. The average cSP was calculated from 12 trials. The iSP indicates an inhibitory influence of the stimulated M1 on the opposite M1, mediated at least in part via the corpus callosum. TMS was applied to the ipsilesional hemisphere at 80% MSO while the participant maintained voluntarily hand contraction as above. The iSP was determined in FDI EMG between 30 and 80 ms post stimulus by detecting the region below one third of the average root mean square amplitude of pre-trigger EMG (25). The iSP duration and area were identified from the average of 12 rectified EMG traces, as was the persistence or number of trials that produced an iSP (26).

### Statistical Analysis

To examine the relationship between hand function measures and the other independent variables, parametric and non-parametric tests were performed using SPSS (V.19, IBM) dependent on the normality distribution of the data. Tests included correlation analysis using Pearson *r* or Spearman rho; analysis of variance (ANOVA or Kruskal–Wallis) tests, with multiple comparison post tests and independent sample tests using Mann Whitney. A stepwise multiple linear regression analysis determined which independent variables (Type of lesion, LI, FAAI) were associated with clinical upper limb function (MUUL). The level of significance was set at α = 0.05 for all analyses. Means and standard deviations are reported unless otherwise stated.

## Results

### Upper limb function and classification

Table 1 summarizes the individual and group mean clinical assessment scores. The mean MUUL score was 80% (range 41–100%), with a strong correlation found between the MUUL and other capacity based upper limb function measures (Box Block: *r* = 0.81; Bimanual: *r* = 0.83; *p* < 0.0001). There was a weak association between MUUL and self-reported difficulty in performing upper limb daily activities determined by the Abilhands (*r* = 0.42, *p* = 0.05). Higher performances on all tasks were found in the MACS level I group [ANOVA: MUUL: *F*(1, 19) = 31.05, *p* < 0.001; Box Block: *F*(1, 19) = 23.8, *p* < 0.0001; Bimanual: *F*(1, 19) = 13.7, *p* = 0.0001; Abilhands: *F*(1, 19) = 7.03, *p* = 0.02]. Six participants had Grade 2 evidence of slight mirror movements during finger tapping, opposition, and hand grip tasks (15).

**Table 1:** **Participant characteristics**.

No.	Age	Sex	Hemi	Lesion	fMRI	DTI	TMS		Upper limb function measures					
					LI	FAAI	MEP present affected hand IL stim CL stim		MACS	Mirror	MUUL (%)	Box block	Bimanual tasks	Abilhands (logits)
1	18	F	R	PV	−0.96	0.04	Y	N	I	–	100	45	30	6.68
2	18	F	R	PV	−1.00	−0.02	n/a	n/a	I	–	95	38	30	5.04
3	16	M	R	PV	−0.26	0.07	Y	N	I	–	98	45	30	6.68
4	12	M	R	PV	−0.80	0.06	n/a	n/a	I	–	98	35	30	6.68
5	22	F	L	PV	−1.00	0.18	n/a	n/a	I	–	86	24	23	6.68
6	13	M	L	PV	−1.00	0.00	Y	N	I	–	99	46	30	5.04
7	16	M	L	PV	−1.00	0.21	n/a	n/a	I	–	92	18	16	6.68
8	16	F	R	PV	−1.00	0.01	Y	N	I	–	100	29	30	6.68
9	14	M	R	PV	−1.00	−0.02	Y	N	I	–	100	45	30	6.68
10	18	F	R	C/SC	−1.00	0.69	n/a	n/a	II	–	56	3	20	1.76
11	12	M	L	C/SC	−1.00	0.03	Y	Y	II	–	64	17	12	3.9
12	13	M	R	C/SC*	1.00	0.18	N	Y	II	Y	71	19	22	0.51
13	15	M	L	C/SC*	−1.00	n∕a	n/a	n/a	II	–	41	4	10	6.68
14	13	M	L	C/SC	−0.05	0.42	N	Y	II	–	46	13	16	3.51
15	12	F	L	C/SC	−1.00	−0.01	n/a	n/a	II	Y	70	14	22	2.89
16	12	F	L	C/SC	1.00	n∕a	n/a	n/a	II	Y	51	6	19	3.9
17	21	M	L	MAL	1.00	0.11	N	Y	I	Y	89	19	23	3.51
18	14	F	L	MAL	−0.54	0.11	Y	Y	II	Y	74	16	18	5.04
19	13	F	R	MAL	−1.00	0.07	Y	N	II	–	93	20	30	1.96
20	17	M	R	MAL	1.00	0.31	N	Y	II	Y	79	18	20	6.68
Mn	15	–	–	–	−0.50	0.15	–	–	–		80	24	23	4.86
SD	3	–	–	–	0.79	0.19	–	–	–		20	14	7	2.00
Min	12	–	–	–	−1.00	−0.02	–	–	–		41	3	10	0.51
Max	22	–	–	–	1.00	0.69	–	–			100	46	30	6.68

*Hemiplegia (R) right; (L) left = affected upper limb*.

Lesion: PV, periventricular; C/SC, cortical/subcortical; MAL, malformation; *evidence of bilateral lesions.

*LI, laterality index derived from functional MRI*.

*FAAI, fractional anisotrophy asymmetry index derived from diffusion-weighted imaging*.

*MEP (IL), motor-evoked potentials in FDI muscle of affected upper limb, from ipsilesional (IL) transcranial magnetic stimulation*.

*MEP (CL), motor-evoked potentials in FDI muscle of affected upper limb, from contralesional (CL) transcranial magnetic stimulation*.

*(Y) Yes, present; (N) No, absent; n/a, not applicable – did not have TMS due to contraindications*.

*MACS, Manual Ability Classification System*.

*MUUL, Melbourne assessment of unilateral upper limb function (%)*.

*Mirror = mirror movements, Y = grade 2 presence of slight mirror movement (15)*.

*Mn, mean; SD, standard deviation; Min, minimum; Max, maximum*.

### Classification of lesion

Lesion characteristics are summarized in Table 1. All participants completed the scanning protocol for the purposes of lesion characterization. There was a difference in upper limb function scores dependent on lesion classification [ANOVA, *F*(2, 17) = 42.48, *p* < 0.0001]. Participants with C/SC lesions had lower mean MUUL (mean MUUL = 57 ± 11%) compared to those participants with PV or MAL (mean MUUL 96 ± 5% and 84 ± 9%, respectively). Participants with C/SC lesions had more impaired upper limb function than the other lesion types, as measured by Box and Block [ANOVA, *F*(2, 17) 20.10, *p* < 0.001], Bimanual tasks [ANOVA, *F*(2, 17) 8.60, *p* < 0.001], and Abilhand-Kids questionnaire [ANOVA, *F*(2, 17) 8.11, *p* < 0.001].

### Functional MRI

**Figure 1:**
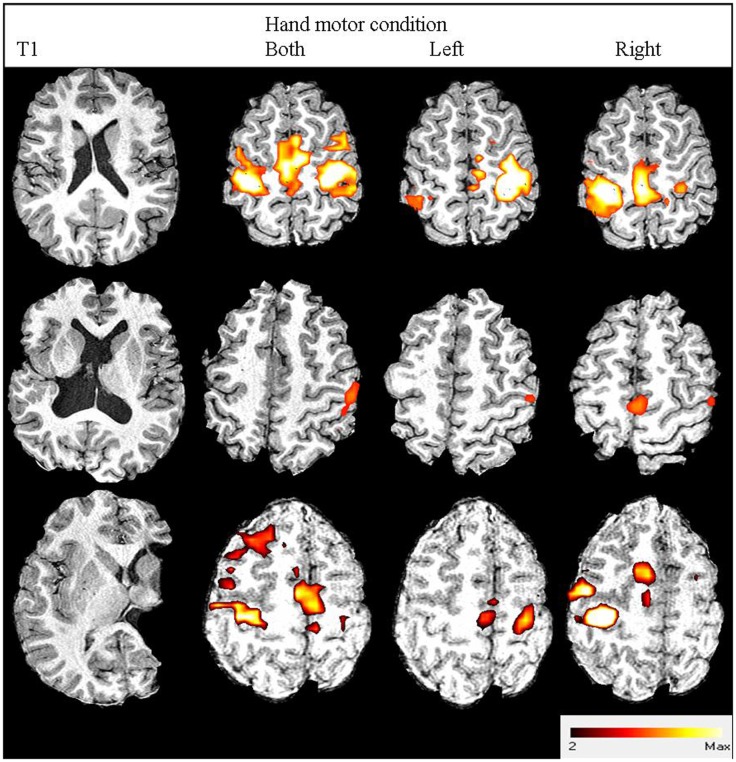
**Three participants’ images from structural and functional MRI**. From left to right: anatomical T1-weighted image; fMRI contrast results thresholded to *z* > 2.3: both hands active condition; left hand active condition; right hand active condition. From top to bottom: participant no. 19 with right hemiplegia and high hand function (93% MUUL) showing normal contralateral (M1/S1, PMd, PMv) and medial (SMA) activation; Participant no. 20 with right hemiplegia and average hand function (79% MUUL) showing a shift in fMRI activation lateralized to CL hemisphere for the affected right hand condition: participant no. 13 with left hemiplegia and poor hand function (41% MUUL) showing less activation than Participant no. 19 but with some remaining contralateral organization.

Performing the fMRI task with the affected hand revealed the expected BOLD activation primarily around the “hand knob” of the precentral gyrus of the ipsilesional hemisphere for 16 participants (27). However, four participants had motor area activation predominantly within the CL hemisphere. Figure 1 shows statistical parametric maps of three participants with distinct activation profiles.

During affected hand movement, the mean LI was −0.53 (range −1.0 to 1.0) for primary and pre motor cortex (Table 1) and 0.00 (range −1.0 to 1.0) for supplementary motor area, with a negative LI indicating greater activity in the appropriate ipsilesional hemisphere. Participants 12, 16, 17, and 20 had activity lateralized toward the CL hemisphere (LI = 1.00). Note, these four participants also all had Grade 2 or slight mirror movements detected on clinical assessment. Participant 14 had near symmetrical activity (LI = −0.05). LI was not associated with any measure of upper limb function (MUUL: *r* = −0.25, *p* = 0.20) or lesion classification [ANOVA, *F*(2, 17) = 0.26, *p* = 0.76].

### Diffusion-weighted MRI

DW–MRI data from two participants (no. 13 and 16) were not usable because of excessive head motion. The mean FA of the ipsilesional (affected) hemisphere PLIC was 0.45 ± 0.14 and in the unaffected hemisphere 0.56 ± 0.08. The group average FAAI was 0.15, range −0.02 to 0.69 (Table 1), with the positive FAAI indicating a reduced anisotropy in the ipsilesional PLIC compared to the CL side. There was a strong association between FAAI and upper limb function assessments (MUUL: *r* = −0.67, *p* = 0.002; Box Block: *r* = −0.65, *p* = 0.003; Bimanual tasks: *r* = −0.49, *p* = 0.03). Participants with better function had more symmetrical FA between the ipsilesional and CL PLIC. FAAI was not strongly related to the BOLD LI values (*r* = 0.24, *p* = 0.30), although, there was a trend for participants with a shift in fMRI activity to the CL hemisphere to have greater asymmetry in FAAI. There was no difference in the FAAI based on lesion classification group [ANOVA, *F*(2, 15) = 2.30, *p* = 0.13], although there was a trend for greater asymmetry in participants with cortical and subcortical lesions (mean FAAI = 0.26 for C/SC lesions; 0.15 for MAL; 0.06 for PV).

### Transcranial Magnetic Stimulation

**Figure 2:**
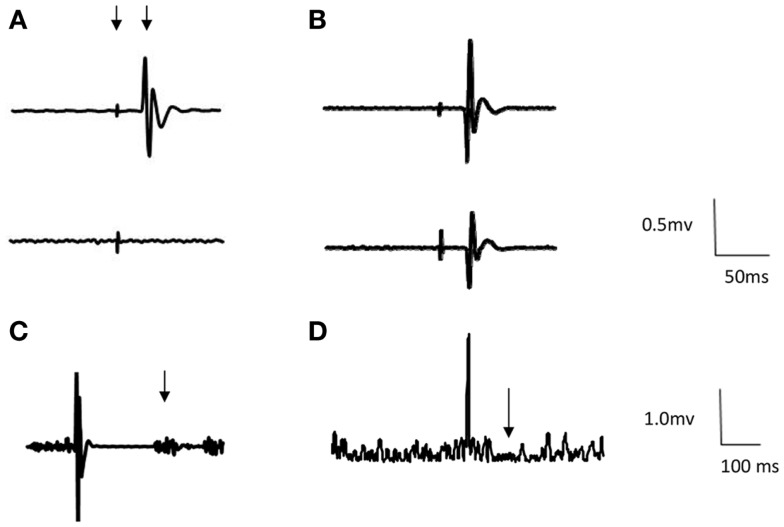
**Representative first dorsal interosseous (FDI) EMG traces from two participants**. Arrows indicate stimulus artifact, MEP [example **(A)**], return of voluntary EMG [example **(C)**], and ipsilateral SP [example **(D)**]. **(A)** Example of appropriate response to TMS to the contralesional hemisphere. Top trace = unaffected FDI and bottom trace = affected FDI (Participant no. 1). **(B)** Example of bilateral response to TMS to contralesional hemisphere. Top trace = unaffected FDI and bottom trace = affected FDI (Participant no. 17). **(C)** Example of cortical silent period following ipsilesional stimulation (Participant no. 1). Traces are averages of 12 sweeps. **(D)**. Example of ipsilateral SP following stimulation ipsilesional hemisphere (Participant no. 1). Traces are rectified averages of 12 sweeps.

Eight participants could not have TMS due to contraindications (i.e., history of epilepsy). Of the 12 tested, all had MEPs in the unaffected FDI from TMS of the CL M1. Mean RMT was 55.3 ± 14.2% MSO with mean MEP latency in the unaffected FDI of 23.0 ± 2.0 ms (Table 2). Six of these participants showed atypical MEPs in FDI bilaterally in response to TMS of the CL M1 (Figure 2). MEP latency of affected FDI was 23.6 ± 1.9 ms.

**Table 2:** **Mean rest motor threshold and MEP latency (120% MSO)**.

Hemisphere stimulated	Rest motor threshold % (SD)	Mean MEP latency ms (SD)
Contralesional	55.3 (14.2)	Unaffected hand: 23.0 (2.0) *n* = *12*
		Affected hand: 23.6 (1.9) *n* = 6
Ipsilesional	70.8 (19.8)	Affected hand: 23.4 (2.0) *n* = *8*

**Inhibitory function:**			

**Hemisphere stimulated**	**Mean cSP duration ms (SD)**	**Mean iSP persistence/12 (range)**	**Mean iSP duration ms (SD)**

Contralesional	Unaffected hand: 141 (56) *n* = *12*	–	–
	Affected hand: 115 (64) *n* = *3*	
Ipsilesional	Affected hand: 86 (47) *n* = *7*	8 (4–11)*n* = *7*	22.7 (5.4) *n* = *7*

*SD, standard deviation; MEP, motor-evoked potentials; cSP, cortical silent period; iSP, ipsilateral silent period*.

*Persistence = number of trials out of 12 where silent period present*.

Transcranial magnetic stimulation of the ipsilesional M1 yielded MEPs in the affected FDI of 8 participants, with a mean RMT 70.8 ± 19.8% MSO and MEP latency of 23.4 ± 2.0 ms (Table 2). Two participants had MEPs in the affected FDI with both ipsilesional and CL stimulation. Four participants had no MEPs in the affected FDI with ipsilesional M1 stimulation.

There was a positive trend in the relationship between the functional integrity of corticomotor pathways and measure of hand function (Kruskal–Wallis, Bimanual tasks *p* = 0.01; MUUL *p* = 0.07), with higher median MUUL and bimanual task scores for participants with normal contralateral pathways compared to those with no MEPs on ipsilesional stimulation or bilateral MEPs with CL stimulation.

All 12 participants had a cSP from CL stimulation (duration 141 ± 56.3 ms), with three participants having bilateral cSP present (duration 115 ± 63.8 ms) and correspondingly no cSP from ipsilesional stimulation (Figure 2). In total, only seven participants had a cSP from ipsilesional stimulation. For these participants mean cSP was 86 ± 46.8 ms. An association between the presence of CL and ipsilesional cSP and upper limb function (Mann Whitney, *p* = 0.02) was found, with higher function (median MUUL 99%) in those with cSP present from both hemisphere stimulation (Table 2). However, there was no association between the duration of CL cSP and upper limb function measures (MUUL, rho = 0.1).

An iSP was obtained in seven participants, with a mean duration of 22.7 ms (range 17–33 ms) and mean percentage inhibition of 15% (range −20 to 79%) (Figure 2). An iSP was not present in the four participants with no ipsilesional pathways, as well as two other participants. There was an association between the amount of iSP inhibition and clinical measures, with greater function in those with more effective inhibition (Kruskal–Wallis *p* < 0.001; post-test MUUL *p* = 0.001; Bimanual *p* = 0.05).

### Relationship with upper limb function

Fractional anisotropy asymmetry index was the best indicator of clinical upper limb function, explaining 41% of the variance for MUUL (*R*^2^ = 0.41, *F* = 11.3, *p* = 0.004). The addition of lesion type further increased predictive power (*R*^2^ = 0.57, *F* = 9.8, *p* = 0.02).

## Discussion

The novel findings of this study are that in unilateral CP intracortical and interhemispheric inhibitory measures vary depending on the structural integrity of descending motor pathways. Generally, participants with intact intracortical and interhemispheric inhibition had higher upper limb function. Understanding the association between upper limb function, motor cortex function, and motor pathway integrity may assist in the targeting and development of novel upper limb rehabilitation strategies in youth with unilateral CP (4).

This is one of the first studies of unilateral CP to describe M1 inhibitory function by evaluating the cortical (cSP) and ipsilateral (iSP) silent periods. Silent period measures reflect activity of GABA_B_-ergic cortical inhibitory mechanisms (24). In neurologically unimpaired individuals, cSP duration can be greater than 200 ms (24). In this study, the duration of the cSP was less than these previously reported values in both the CL (141 ± 56 ms) and ipsilesional hemispheres (86 ± 42 ms), with only 7 out of 12 participants showing a cSP with ipsilesional stimulation. These results are similar to a previous study in a CP sample that found reduced cSP durations in the lower limb in children with spastic diplegia (28).

The iSP is mediated, at least in part, by transcallosal pathways and reflects the ability of the ipsilesional hemisphere to inhibit the CL hemisphere through GABA-ergic interneurons (24). The iSP in a neurologically intact population has been shown to have an average duration of between 25 and 33 ms (26, 29). In the current study, the average iSP duration was 23 ms, but there was wide variation in the amount of inhibition and 5 of the 12 participants assessed did not show any iSP. In the adult stroke population, a reduced interhemispheric inhibition from ipsilesional to CL primary motor cortex is common, affecting the balance of excitability between the hemispheres (30). Furthermore, interventions that restore balance in hemispheric excitability by modulating intracortical and interhemispheric inhibition have been associated with better outcomes (30). Enhancing interhemispheric inhibition is a potential mechanism that could be targeted in unilateral CP. It remains to be determined if this would improve outcomes for these participants.

The three main types of brain lesions in the current sample (PV; MAL; cortical/subcortical), represent the most frequent structural lesions described in unilateral CP (16). In our study, upper limb function was related to lesion type, as observed previously, where individuals with PV lesions had better hand function than individuals with cortical, subcortical, or acquired lesions (16, 31). Staudt and colleagues also found that individuals with larger PV lesions and those with lesions developing late within the third trimester (predominantly cortical and deep gray matter), had worse hand function (8). In contrast, others have found no correlation between lesion type and motor impairment (32). This difference might be partially explained by the use of non-standardized measures of hand function in previous studies. Lesion volume has also been previously shown to impact function (5), though this was not explored in this current study, in which the focus was on corticospinal tract integrity specifically.

Among the five participants who showed motor pathway reorganization, there was a trend toward more impaired upper limb function, similar to previous findings (8). fMRI data indicated these participants had a shift in the cortical control of the affected hand toward the CL hemisphere. These individuals had either a cortical/subcortical lesion (*n* = 3) or malformation (*n* = 2) and a spread of upper limb function mostly across the lower range. Holmstrom et al. (33) also found the lowest levels of hand function in individuals with reorganization to the CL hemisphere (*n* = 5) (33). However, there was again variation in ability across these individuals indicating that there may be potential for some individuals with CL reorganization to attain a good level of upper limb function. In general, the small number of participants with CL reorganization is a limitation in both our work and previous studies and remains a topic of further enquiry (7, 9).

Hemisphere laterality of motor activity, as determined from fMRI, was not related to hand function measures for the participants in this study, potentially reflecting the variability in the group described above. Functional MRI is a useful tool in detecting corticomotor re-organization and our results confirm earlier studies that contralateral control is not a default mode of upper limb function in this population (7, 34). However, TMS and motor-evoked potentials may offer a more in depth assessment of corticomotor function, specifically within M1.

Interestingly, descending white matter pathway integrity, as determined by asymmetry of FA measurements in the PLIC was strongly related to the upper limb functional measures. This is consistent with Holmstrom et al. (35) who also found mean FA values in the PLIC of children with unilateral CP correlated to the Box and Block measure of gross motor hand function (35). In children with CP, PLIC FA may be able to provide information that anatomical MRI alone cannot provide, such as a quantitative threshold level to indicate whether sufficient motor pathways are intact for particular interventions (36). As such, adopting DW–MRI provides knowledge of the specific pathophysiology and may be useful to guide treatment planning away from a “one-size fits all” treatment approach (37).

As a progression from this, recent work has found variable outcomes in the association of MRI findings and efficacy of CIMT in children with unilateral CP. Three recent small studies found no relationship between the amount of improvement following a CIMT program and brain lesion characteristic or organization (38–40), with three other studies finding imaging information did have a predictive ability for the efficacy of a CIMT program (4, 41, 42). However, authors caution at this early stage that generalization of results is not possible due to the small sample sizes (39).

### Limitations

Unilateral CP is characterized by a high degree of heterogeneity in terms of lesion type making it difficult to generalize findings from individual studies; the future use of meta-analyses may allow stronger conclusions. In particular, the TMS results in this study were taken from a smaller sample of just 12 participants. This study was unique in that it used a wide range of standardized upper limb functional measures and classifications to relate to neuroimaging and neurophysiological measures. This supports the recommendation from a recent systematic review on use of DW–MRI in CP on the inclusion of standardized functional measures, such as the Melbourne UUL, to allow for future meta-analysis (43).

Both fMRI and some TMS techniques are more appropriate in the older child, due to active participation and tolerance of the procedure. However, quantitative assessment using DW–MRI has potential to be added to routine structural MRI in younger children. However, the ability to complete standardized analysis of this information to fit into a clinical setting remains a challenge (43). No attempt was made to determine intra or inter-rater reliability of the imaging-based measures, which is a potential limitation.

## Conclusion

These results indicate that DW–MRI and TMS measures provide a useful addition to standard MRI and relate to upper limb function. Such techniques may help in individualizing therapy, based on characteristics such as motor pathway integrity and reorganization profile. This paper serves to improve our understanding on the inter and intra hemispheric inhibitory mechanisms operating in children with unilateral CP, with potential for these mechanisms to become a target of intervention, similar to what is now being explored in the adult stroke literature.

## Conflict of Interest Statement

The authors declare that the research was conducted in the absence of any commercial or financial relationships that could be construed as a potential conflict of interest.

## Abbreviations

BOLD, blood oxygenation level-dependent; C/SC, cortical and subcortical lesion; CIMT, constraint-induced movement therapy; CL, contralesional hemisphere opposite to lesion; CP, cerebral palsy; cSP, contralateral silent period; DW-MRI, diffusion-weighted MRI; ECR, extensor carpi radialis; EMG, electromyography; FA, fractional anisotropy; FAAI, fractional anisotropy asymmetry index; FDI, first dorsal interosseous; fMRI, functional MRI; IL, ipsilesional hemisphere on side of lesion; iSP, ipsilateral silent period; LI, laterality index; M1, primary motor cortex; MACS, manual ability classification system; MAL, malformations; MEP, motor-evoked potential; MRI, magnetic resonance imaging; MSO, muscle stimulator output; MUUL, Melbourne assessment of unilateral upper limb function; PLIC, posterior limb of internal capsule; PV, periventricular; RMT, resting motor threshold; ROI, region of interest; TMS, transcranial magnetic stimulation.
